# Genetic variation in transpiration efficiency and relationships between whole plant and leaf gas exchange measurements in *Saccharum* spp. and related germplasm

**DOI:** 10.1093/jxb/erv505

**Published:** 2015-11-30

**Authors:** Phillip Jackson, Jaya Basnayake, Geoff Inman-Bamber, Prakash Lakshmanan, Sijesh Natarajan, Chris Stokes

**Affiliations:** ^1^CSIRO, Australian Tropical Science Innovation Precinct, Private Mail Bag PO, Aitkenvale, QLD 4814, Australia; ^2^Sugar Research Australia Limited, PO Box 117, Ayr, QLD 4807, Australia; ^3^Sugar Research Australia Limited, 50 Meiers Road, Indooroopilly, QLD 4068, Australia; ^4^Crop Science Consulting, 33 Tamarind Street, Kirwin, QLD 4817, Australia

**Keywords:** conductance, germplasm, photosynthesis, sugarcane, transpiration efficiency, water use efficiency.

## Abstract

Fifty-one genotypes of sugarcane (*Saccharum* spp.) or closely related germplasm were evaluated in a pot experiment to examine genetic variation in transpiration efficiency. Significant variation in whole plant transpiration efficiency was observed, with the difference between lowest and highest genotypes being about 40% of the mean. Leaf gas exchange measurements were made across a wide range of conditions. There was significant genetic variation in intrinsic transpiration efficiency at a leaf level as measured by leaf internal CO_2_ (Ci) levels. Significant genetic variation in Ci was also observed within subsets of data representing narrow ranges of stomatal conductance. Ci had a low broad sense heritability (H_b_ = 0.11) on the basis of single measurements made at particular dates, because of high error variation and genotype × date interaction, but broad sense heritability for mean Ci across all dates was high (H_b_ = 0.81) because of the large number of measurements taken at different dates. Ci levels among genotypes at mid-range levels of conductance had a strong genetic correlation (−0.92 ± 0.30) with whole plant transpiration efficiency but genetic correlations between Ci and whole plant transpiration efficiency were weaker or not significant at higher and lower levels of conductance. Reduced Ci levels at any given level of conductance may result in improved yields in water-limited environments without trade-offs in rates of water use and growth. Targeted selection and improvement of lowered Ci per unit conductance via breeding may provide longer-term benefits for water-limited environments but the challenge will be to identify a low-cost screening methodology.

## Introduction

In sugarcane (*Saccharum* spp.), as with other crop species, the ability of a crop to produce high biomass and yield per unit of available water is potentially important in affecting profitability and yield, in both irrigated and rainfed production systems (Robertson and Muchow, 1994; Inman-Bamber and McGlinchey, 2003; Inman-Bamber and Smith, 2005; Basnayake *et al.*, 2012). Like many other crops, most rainfed sugarcane crops throughout the world experience some level of water stress, sometimes severe. In irrigated crops, there is increasing concern about the amount and efficiency of water use owing to the rising costs of applying water, limited availability of irrigation water, and competing environmental concerns.

The concept of water use efficiency is depicted in the use of various formats of the simple framework:

$$\begin{array}{l} \text{Yield }=\text{ water transpired x transpiration }\\ \text{efficiency }\left(\text{TE}\right)\text{ x harvest index }\left(\text{Eqn1}\right) \end{array}$$

This framework or slight modifications of it have been widely used among researchers to conceptualize and interpret causes of yield variation in water-limited environments (e.g. Passioura, 1977; Condon and Richards, 1993; Richards *et al.*, 2002). This has proven to be useful in interpreting results investigating variation in crop growth. According to this framework, growth in water-limited environments may be increased if there is more transpiration (i.e. more water accessed and acquired by roots and transpired) or if there is more biomass produced per unit water transpired.

C_4_ plants like sugarcane are well known to have generally higher transpiration efficiency (TE) than C_3_ species (e.g. Osmond *et al.*, 1982; Ghannoum, 2009). An important feature of C_4_ plants is a modification of the ancestral C_3_ pathway involving a mechanism effectively concentrating CO_2_ into specialized bundle sheath cells for fixation by Rubisco and the rest of the C_3_ cycle. This leads to higher levels of photosynthesis at lower levels of leaf intercellular CO_2_ (Ci) than C_3_ plants and suppression of photorespiration (Hatch, 1987). Compared with C_3_ photosynthesis, the abrupt saturation of photosynthesis at relatively low levels of Ci in C_4_ plants facilitates high photosynthesis at relatively low levels of stomatal conductance, and hence higher TE (Ghannoum 2009).

Apart from the general difference between C_4_ and C_3_ plants, it is also apparent from many reports that significant and potentially economically important genetic variation in TE exists within many crop species, including C_4_ species (e.g. Hammer *et al.*, 1997; Henderson *et al.*, 1998; Mortlock and Hammer, 1999), although no survey of variation in a broad range of sugarcane germplasm has been reported to our knowledge. 

Given the apparent genetic variation in TE, this trait has also been widely considered as a potential objective in plant breeding programmes in a range of important crops. However, problems with the use of TE as a selection criterion in breeding programmes have been widely experienced in practice, and discussed extensively (e.g. Condon *et al.*, 2004; Blum, 2009; Sinclair, 2012). One complication is a possible negative genetic covariance occurring between the first two components in Equation 1 (i.e. water transpired and TE), at least in some environments. This negative relationship may arise because genotypes with lower stomatal conductance—due, for instance, to genetic differences in leaf anatomy or sensitivity to mild water stress or other environmental cues (and, in the absence of photosynthesis, impairment due to more severe stress)—have lower Ci and therefore a greater CO_2_ partial pressure diffusion gradient between the interior of the leaf and the external air. This in turn increases TE, which is directly proportional to the ratio between the gradient in CO_2_ concentration from the outside to the inside of the leaf, and the gradient in water vapour concentration from the inside to the outside of the leaf (Condon *et al.*, 2004) as follows:

$$\text{TE}\sim \left({\text{C}}_{a}-{\text{C}}_{i}\right)/\left({\text{W}}_{i}-{\text{W}}_{a}\right)$$

where C_i_ and C_a_ are the CO_2_ concentrations inside and outside the leaf respectively, and W_i_ and W_a_ are the concentrations of water vapour inside and outside of the leaf respectively. Given the relationship depicted in Equation 1, reduced transpiration arising from intensive selection for increased TE via lower conductance in some environments may offset benefits in yield arising from higher TE. In some cases where genetic variation for transpiration is greater than for TE, strong selection pressure for high TE may even be counterproductive, resulting in reduced biomass (Condon *et al.*, 2004).

Sinclair (2012) and others have also highlighted the dependency of TE on the atmospheric vapour pressure deficit (VPD; which affects W_a_ in Equation 2). Thus, genetic variation in the response of conductance to temporal variation in VPD, either within diurnal cycles or across a longer time frame, will also give rise to variation in TE and induce a negative genetic covariance between transpiration and TE (e.g. Fletcher *et al.*, 2007).

TE is therefore not associated with a single simple response, or a constant for any particular genotype, but is a complex of several underlying mechanisms. These mechanisms may need to be broken down into subcomponent traits that can be measured reliably and repeatably before useful applications in breeding programmes may be developed (Condon *et al.*, 2004). Based on past research, we propose a general hypothesis that attaining higher yields by improving TE in crop improvement programmes requires an understanding of the extent of genetic variation in the key mechanisms causing the observed variation, and that selection in breeding programmes is best applied to subcomponents of TE, rather than selection for TE *per se*. Genetic variation in TE may arise through three higher level mechanisms: (i) genetic variation in the response of conductance to changes in VPD, (ii) genetic variation in conductance independent of VPD causing variation in Ci (Equation 2), and (iii) genetic variation in photosynthesis rate, and thus Ci, for any given level of conductance (Condon *et al.*, 2004; Gilbert *et al.*, 2011). In order to predict the value of higher TE in improving yield under any particular group of targeted production environments, it is important to consider and predict the impact of each of these mechanisms separately because of potentially varying impacts and genetic correlations with transpiration. Genetic variation associated with mechanisms (i) and (ii) above may lead to negative genetic correlations between rate of crop transpiration and TE (as discussed above), which under different environments may lead to either positive or negative net impacts on final yield. These impacts may be variable and difficult to predict depending on changes in timing and severity of water stress across the entire course of a crop growth cycle. By contrast, increased TE arising from higher photosynthesis rates at any given level of conductance (giving rise to reduced Ci levels within any given range of conductance) would be expected to have generally positive effects.

In this paper we first investigate genetic variation in a wide range of sugarcane and related germplasm for TE, which represents the first reported survey for this crop to our knowledge. This variation is then related to measures of leaf level intrinsic TE, as measured by Ci. The results are used to more generally consider approaches to screening germplasm in breeding programmes that aim to develop improved TE without undesirable indirect selection for reduced transpiration and slower growth rates.

## Methods

### Genetic material

Fifty-one clones representing a range of important modern sugarcane cultivars from Australia and some other countries, important parental material in the Australian sugarcane breeding programme, and a range of species within the so-called *Saccharum* complex (Mukherjee, 1957; Daniels and Roach, 1987) were chosen to represent a wide range of genetic diversity potentially available for sugarcane improvement programmes (Table 1). Sugarcane breeding programmes throughout the world share common ancestors (Arceneaux, 1967) and therefore results from the Australian breeding programme may be considered to be broadly applicable to sugarcane cultivars and parental material elsewhere.

**Table 1.** List of clones examined, and species designation of each

Clone	Species/designation^a^	Whole plant TE (g/L)	Ci (at 0.2–0.3 conductance) (µmol mol^−1^)	Ci (average) (µmol mol^−1^)	Conductance (mol m^−2^ s^−1^)
A_MAURITIUS	*S. officinarum*	5.71	163	168	0.214
BADILA	*S. officinarum*	7.30	130	143	0.238
BLACK_FIJI	*S. officinarum*	7.56	134	144	0.206
BLACK_TANNA	*S. officinarum*	6.24	165	158	0.215
BURMA	*S. spontaneum*	6.16	169	163	0.157
CYC96-40	*S. officinarum x Erianthus arundinaceus*	7.53	158	134	0.183
FIJI_62	*S. officinarum*	7.04	136	133	0.229
IJ76-315	*S. officinarum*	7.23	148	162	0.285
IJ76-357	*E. arundinaceus*	6.84	115	136	0.228
IJ76-365	*E. arundinaceus*	7.46	114	143	0.24
IJ76-381	*E. arundinaceus*	7.96	130	153	0.267
IJ76-394	*E. arundinaceus*	8.63	149	157	0.23
IJ76-411	*S. robustum*	6.77	127	133	0.254
IK76-71	*S. spontaneum*	6.85	155	170	0.45
IS76-196	*S. spontaneum*	5.80	195	171	0.329
JAMAICA_RED	*S. officinarum*	5.91	174	170	0.214
KORPI	*S. officinarum*	7.50	143	142	0.178
LOUISIANA_ST	*S. officinarum*	6.70	130	163	0.214
MANDALAY	*S. spontaneum*	7.55	156	156	0.411
N29	Commercial cultivar	6.34	146	151	0.245
NG28-101	*S. spontaneum*	6.82	142	146	0.386
NG51-99	*S. officinarum*	6.64	178	166	0.208
PINDAR	Commercial cultivar	6.12	159	175	0.227
Q20	*S. officinarum*	6.99	135	163	0.252
Q28	Commercial cultivar	6.12	162	162	0.289
Q119	Commercial cultivar	6.66	132	143	0.286
Q183	Commercial cultivar	6.67	163	160	0.22
Q200	Commercial cultivar	7.22	137	137	0.201
Q208	Commercial cultivar	7.47	145	157	0.22
KQ228	Commercial cultivar	5.95	143	144	0.236
Q229	Commercial cultivar	6.70	128	134	0.256
MQ239	Commercial cultivar	7.57	147	135	0.19
Q240	Commercial cultivar	6.49	145	149	0.264
Q247	Commercial cultivar	6.88	130	134	0.202
Q252	Commercial cultivar	6.21	149	159	0.239
Q253	Commercial cultivar	7.34	121	143	0.179
Q256	Commercial cultivar	7.45	139	138	0.206
QA01-5267	Commercial parent	6.40	138	149	0.222
QA04-1448	Commercial parent	6.94	158	158	0.228
QBYC05-20735	Commercial hybrid × *S. spontaneum*	7.00	150	162	0.246
QBYC05-20853	Commercial hybrid × *S. spontaneum*	6.48	149	150	0.221
QBYN04-10951	*S. officinarum* × *S. spontaneum*	6.61	133	143	0.285
QC91-580	Commercial parent	6.87	141	154	0.211
QN66-2008	Commercial parent	8.28	128	146	0.162
QN04-121	Commercial parent	6.00	128	137	0.304
QN04-1643	Commercial parent	6.25	146	158	0.214
QS00-486	Commercial parent	6.80	145	144	0.232
QS01-1078	Commercial parent	7.21	135	147	0.192
QS04-772	Commercial parent	5.76	165	161	0.226
MEAN		6.86	145	152	0.241
LSD (*P* < 0.05)		1.46	12.7	12.4	0.030

^a^ Commercial cultivars or parents are complex derivatives of *S. officinarum* × *S. spontaneum*.

### Experimental design

The experimental design and methodology used in this study were informed by a prior pilot study on optimizing methodology, including testing different potting mixes, pot sizes, and determining estimates of experimental error variation in key measurements. Based on the latter, we used two replications of each of 51 clones arranged in a randomized complete block design. This configuration maximized the statistic ‘gain from selection’ (Falconer and Mackay, 1996) that incorporates the opposing benefits of increasing numbers of clones tested (to increase selection intensity) versus increasing the number of replicates (to increase broad sense heritability [H_b_]), within the constraints of a fixed number of experimental units (number of clones × number of replicates).

### Growth of plants

Stem pieces (as single-eye setts) of the clones were initially germinated in trays on 1 April 2014 and then transplanted on 5 May 2014 into pots. Pots (24L capacity, 0.3 m deep × 0.35 m diameter) were filled to a uniform overall bulk density with a well mixed 50:50 (by volume) combination of peat moss and sand, with added slow-release fertilizer at approximately 50 g per pot (Osmocote™, 19.4:1.6:5% of N:P:K, respectively, plus micronutrients). A mulch of coarse gravel (about 1cm diameter) about 5cm deep was placed on the surface of each pot to minimize water evaporation directly from the potting mix. Several pots without plants were maintained at field capacity to estimate water loss not due to transpiration. This amount was found to be a negligible proportion (<3%) of water loss from pots with plants over the duration of the experiment.

After transplanting plants from the trays, pots were initially watered sufficiently with an automatic watering system to avoid any water stress for approximately 1 month and any water applied in excess of field capacity was allowed to drain through holes in the bottom of each pot. On the 13 June 2014, the drainage holes were plugged and measured amounts of water were applied to bring all pots to field capacity each day. Field capacity (38% volumetric) was estimated by saturating several pots and then allowing these to drain for 24 hours before measuring moisture content in the potting mix using time-domain reflectometry (TDR) probes inserted into holes in the side of each pot halfway between the top and bottom of the pot.

At approximately 3.00 pm each week day, the moisture level in each pot was measured by insertion of the TDR probe diagonally across the middle section of each pot (through holes in the side of the pot that could be sealed after measurement) and the amount of water to be added to each pot to restore field capacity was calculated and added. Random measurements of spatial variation of moisture content (data not shown) indicated quite uniform moisture content was reached within several hours of watering. Approximately every two weeks, 500 mL of liquid fertilizer (Aquasol™) was added to avoid any nutritional deficiencies. No significant pests or diseases were observed. On weekend days an amount of water was added to each pot corresponding to the average added during the preceding 5 days.

**Fig. 1.**
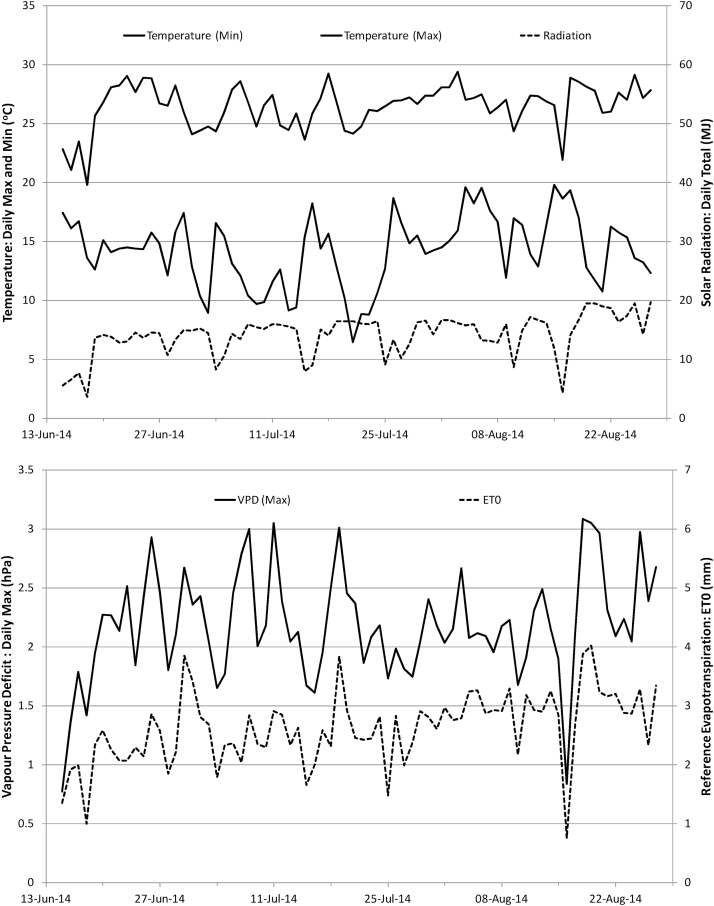
Daily metrics of temperature, radiation, humidity, and evapotranspiration during the measurement period of the experiment.

The experiment was conducted outdoors at the Australian Tropical Science Innovation Precinct, Townsville (19.25°S, 14.81°E) with the pots situated in an unshaded flat area. A weather station was set up on site to monitor temperature, humidity, wind speed and direction, radiation, and rainfall, with several of these parameters shown in Fig. 1.

One rainfall event occurred during the experiment when 68 mm of rain fell over 2 days. On the second day of rain, all pots were unplugged and then allowed to drain for approximately 24 hours. For the purpose of calculating water supplied during these 2 days of rain it was assumed that evapotranspiration from the pots was zero, and that rainfall minus drainage supplied an amount of water to each pot equivalent to the difference between moisture measurements made immediately after and before the rainfall.

Plants were grown until the 25 August 2014, that is, for 16 weeks after transplanting, at which time all pots were harvested over the period between 25 and 27 August 2014.

### Leaf gas exchange measurements

Leaf gas exchange measurements were made using LICOR 6400 instruments two to three times per week on subsets of the pots to characterize clones for stomatal conductance, leaf level photosynthesis, and Ci concentration. Dates of measurement can be seen in Supplementary Table S1 at *JXB* online. A total of 2488 individual leaf measurements were made during the course of the experiment, that is, an average of ca. 50 measurements per clone. Generally, the youngest fully expanded leaf was measured, based on procedures developed in prior studies with sugarcane (Inman-Bamber *et al.*, 2011). Measurement conditions included 2000 μmol quanta m^−2^ s^−1^, 390 ppm CO_2_, and temperature and VPD conditions of the chamber air close to ambient conditions. All parameters were calculated using the manufacturer’s software. Measurements were made between 9.00 am and 3.00 pm and aimed to sample a range of VPD and water stress conditions.

### Whole plant traits

On 13 June 2014, corresponding with the start of water use measurement, the total biomass (shoots and roots) of four additional pots of the clone Q208 was sampled and oven dried (70°C for 3 days). The amount of biomass at this time was proportionally small (<5%) compared with final harvest biomass and the variation in biomass between genotypes at this very early stage of growth was considered insignificant in relation to differences at final harvest. Therefore, any differences between genotypes for starting biomass were assumed to be zero for the purpose of determining biomass production between 13 June 2014 and when the experiment was terminated on 25 August 2014. At this time (and for a period of the next 3 days), all plants in each pot were cut at ground level, leaf area determined, and total aboveground dry biomass measured after oven-drying at 70°C for 3 days. Underground biomass (both root and stem matter) was determined in the following week using a root-washing device that separated roots from potting mix. Some small roots may have been lost during this procedure, but from observation this loss was considered to be a small proportion (<10%) of the total root mass.

TE was estimated for each pot from the ratio of biomass produced from 13 June until final harvest and the total water used by each pot during this period. TE was determined both on the basis of tops alone (i.e. aboveground biomass per water used) and total biomass (i.e. tops + roots biomass per water used).

### Data analysis

Analyses of variance (for individual traits) and covariance (for some pairs of traits) were conducted using the SAS statistical package (version 9.2). For most traits, data for individual pots were used as the basic experimental unit and pot values were analysed as a randomized complete block experimental design, partitioning total variance into that due to blocks, genotypes, and experimental error. For the leaf gas exchange measurements, data obtained were averaged for each pot prior to analyses of variance or covariance. All measurements derived from gas exchange measurements were initially pooled across all dates for analysis, with dates and genotype × date included as factors in the analysis of variance model.

Results for different measurements are presented using statistical parameters routinely used in quantitative genetics (e.g. Falconer and Mackay, 1996), including genetic variance (σ_g_^2^), genetic and error coefficients of variation (CVs) expressed as a percentage (general CV = σ_g_/mean × 100; error CV = σ_e_/mean × 100), and broad sense heritability (i.e. the estimated proportion of variation in observed genotype means due to genetic effects; H_b_ = σ_g_^2^/[σ_g_^2^ + σ_e_^2^/r], where r = number of replicates = 2). For leaf gas exchange measurements made across multiple times, broad sense heritability could be calculated on the basis of different numbers of measurement as: H_b_ = σ_g_^2^/[σ_g_^2^ + σ_gt_^2^/t + σ_e_^2^/tr], where σ_gt_^2^ = variance due to genotype × time interaction, t = number of times of measurement, and tr = total number of measurements of each genotype.

Phenotypic correlations between traits were calculated based on the Pearson correlation coefficient calculated from means of observed traits for each genotype. Genetic correlations between particular pairs of traits were estimated using the standard formula for this statistic (Falconer and Mackay, 1996):

$$\gamma_{g}=\frac{\;Cov_{g(xy)}}{\sqrt{\sigma_{gx}^{2}}\;\sqrt{\sigma_{gy}^{2}}}$$

where *Cov*_*g*(xy)_ is the genetic covariance of the product of two traits or measurements (x and y the two traits being analysed) and *σ* ^*2*^_*gx*_ and *σ* ^*2*^_*gy*_ are the genetic variances of the two traits. Genetic correlations provide an estimate of the correlation between genetic effects for two traits, in contrast to phenotypic correlations, which are a result of the joint effect of correlations between genetic and experimental error effects (the latter may range from positive to negative).

## Results

### Whole plant measurements

There was significant (*P* < 0.05) genetic variation for all traits measured on a whole plant basis (Table 2). Phenotypic values for whole plant TE ranged from 5.7 to 8.6g/L (this range being about 40% of the overall mean). Genetic variation was high for both components of TE—total biomass and water use—and highest for biomass of roots, as indicated by the genetic CVs (Table 2). However, there was proportionally less genetic variation for TE than for the individual components, as indicated by the smaller genetic CV for this trait compared with the two components of this ratio. TE on the basis of total biomass showed proportionally less genetic variation (measured on the basis of genetic CV) than TE on the basis of only tops biomass, as indicated by the larger genetic CV for the latter. Broad sense heritabilities were high (>0.7) for all traits apart from TE on the basis of total biomass. The decrease in whole plant TE on the basis of total biomass was due to the reduced genetic variation in this trait, and in spite of the relatively lower error variation compared with other traits.

**Table 2.** Summary results for key traits

Trait	Mean	GCV (%)	CV (%)	H_b_
Leaf area (cm^2^/pot)	10157	19.8**	28.5	0.49
Biomass of tops (g/pot)	245	33.5***	20.7	0.84
Biomass of roots (g/pot)	137	44***	26.5	0.85
Total biomass (g/pot)	383	36.3***	21.3	0.85
Water use (kg)	57367	38.4***	25.7	0.82
TE tops (g/kg)	4.47	11.1**	9.4	0.74
TE total (g/kg)	6.86	5.8*	10.7	0.37

CV, coefficient of variation; GCV, genetic coefficient of variation; Hb, broad sense heritability; TE, transpiration efficiency. *P < 0.05, **P < 0.01, ***P < 0.001.

**Fig. 2.**
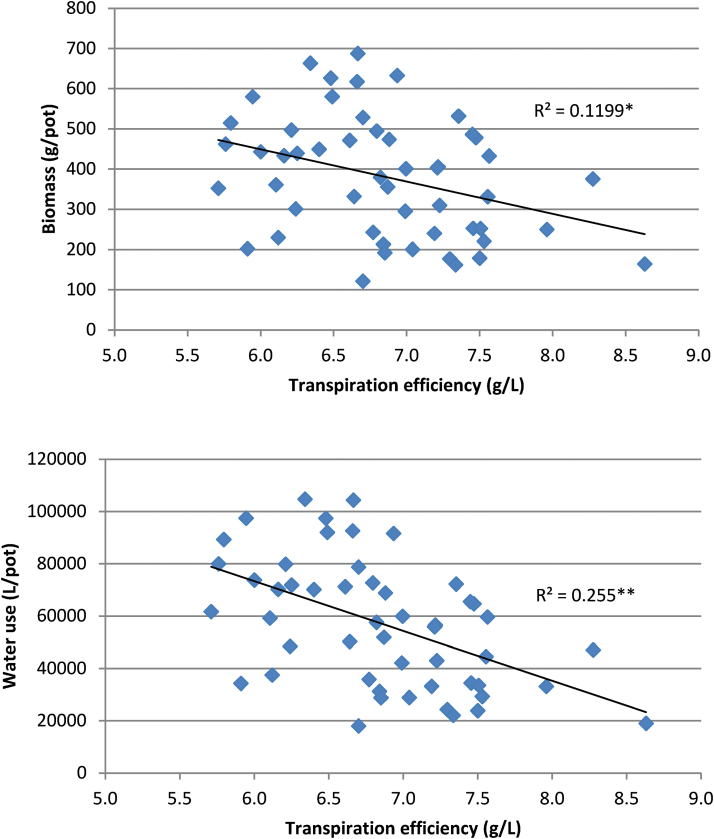
Performance of 51 clones for TE versus total biomass (top, r = −0.35*) and for TE versus water use (bottom, r = −0.50**). *P < 0.05, **P < 0.01.

Phenotypic correlations between genotype means for different traits are shown in Table 3. Water use was tightly related to total biomass (r = 0.98) and leaf area (r = 0.75). The relationships between root to shoot ratio and biomass showed that, on average, high biomass clones partitioned more biomass to roots, although this relationship was not strong (r = 0.41). TE based on tops was moderately (r = 0.72) correlated with TE based on total biomass. TE, based on either plant tops or total biomass, was weakly but negatively correlated with both total biomass and water use (Table 3, Fig. 2).

**Table 3.** Phenotypic correlations (lower diagonal) between different traits

	Leaf area	Tops biomass	Roots biomass	Root:shoot ratio	Total biomass	Water use	TEtops	TEtotal
Leaf area	1.00							
Tops biomass	**0.79**	1.00						
Roots biomass	**0.62**	**0.88**	1.00					
Root:shoot ratio	0.03	0.23	**0.64**	1.00				
Total biomass	**0.74**	**0.98**	**0.96**	**0.41**	1.00			
Water use	**0.75**	**0.96**	**0.94**	**0.39**	**0.98**	1.00		
TEtops	−0.33	**−0.44**	**−0.66**	**−0.69**	**−0.55**	**−0.65**	1.00	
TEtotal	**−0.41**	**−0.36**	−0.30	−0.02	**−0.35**	**−0.50**	**0.72**	1.00

TE is expressed both on the basis of both tops only (TEtops) and total biomass (TEtotal). Correlations with an absolute value >0.33 indicate a statistically significant (*P* < 0.05) linear relationship and are indicated in bold.

### Leaf gas exchange measurements

There was significant (*P* < 0.001) variation due to dates of measurement, genotypes, and genotype × date interactions for the key parameters photosynthesis, conductance, and Ci (Table 4). Variance due to genotype × date interactions was similar in magnitude to variance due to genotype main effects for photosynthesis and conductance, but greater than genotype main effects for Ci. Although error variance components were high (relative to genotype main effects) for all measurements, the large numbers of measurements made provided high broad sense heritabilities (H_b_ > 0.8) if genotype means averaged across all times of measurement were considered (Table 4). However, heritability on the basis of measurements made on one replicate plant at a single time was low (H_b_ ≤ 0.2), due to both high experimental error variance and genotype × date interaction. This indicates there would be low precision in characterizing relative genotype performance on the basis of measurements if measurements were made at only one or a small number of times. The high heritabilities observed in the experiment reported here were possible due to the very large numbers of observations on each clone across different dates.

**Table 4.** Summary statistics from analysis of variance of traits derived from leaf gas exchange measurements

Trait	Mean	Variance components due to:				Broad-sense heritability based on:	
		Dates	Genotypes	Genotype × date interactions	Error	All times	Single time
Photosynthesis (μmol m^−2^ s^−1^)	26.9	39.5	15.09	12.48	50.3	0.93	0.20
Conductance (mol m^−2^ s^−1^)	0.22	0.0015	0.00116	0.000952	0.0062	0.91	0.22
Ci (μmol mol^−1^)	152	143	100.7	281.3	969	0.81	0.11

Broad sense heritabilities calculated on the basis of all times of measurements and on only a single time of measurement are shown. Variation attributed to dates, genotypes, and genotype × date interactions for all traits were statistically significant (*P* < 0.001).

Means for conductance, photosynthesis, and Ci, averaged across all clones, for different dates of measurement are shown in Supplementary Table S1 at *JXB* online. Reasons for variation between different dates may be at least partly attributed to different VPD and air temperatures, with both conductance and photosynthesis being moderately or weakly correlated with VPD (negatively) and temperature (Table 5). Across dates, conductance was positively and reasonably strongly correlated with photosynthesis, as expected, whereas Ci was negatively correlated with photosynthesis but not correlated with conductance (Table 5).

**Table 5.** Correlations between mean conductance, photosynthesis, Ci, VPD, and air temperature across dates

	Conductance	Photosynthesis	Ci	VPD	Temperature
Conductance	1				
Photosynthesis	0.67	1			
Ci	0.19	−0.55	1		
VPD	−0.34	−0.42	0.13	1	
Temperature	0.36	0.28	0.046	0.28	1

Time of day also had significant (*P* < 0.001) impacts on conductance, photosynthesis, and Ci overall, with both conductance and photosynthesis being greatest in the morning (Table 6). By 12.00–2.00 pm, both conductance and photosynthesis dropped proportionally from morning values by a similar amount, so that mean Ci values remained unchanged. After 2.00 pm, conductance remained similar but photosynthesis declined slightly further, giving rise to higher Ci values on average.

**Table 6.** Mean conductance, photosynthesis, and Ci (averaged across all clones and dates) for three different times of day

Time of day	Conductance (mol m^−2^ s^−1^)	Photosynthesis (μmol m^−2^ s^−1^)	Ci (μmol mol^−1^)
10.00am – 12.00pm	0.242	29.1	149.6
12.00pm – 2.00pm	0.216	26.8	149.8
2.00pm – 4.00pm	0.211	24.9	161.0

Values at different times for each attribute are statistically significantly different, *P* < 0.001.

**Fig. 3.**
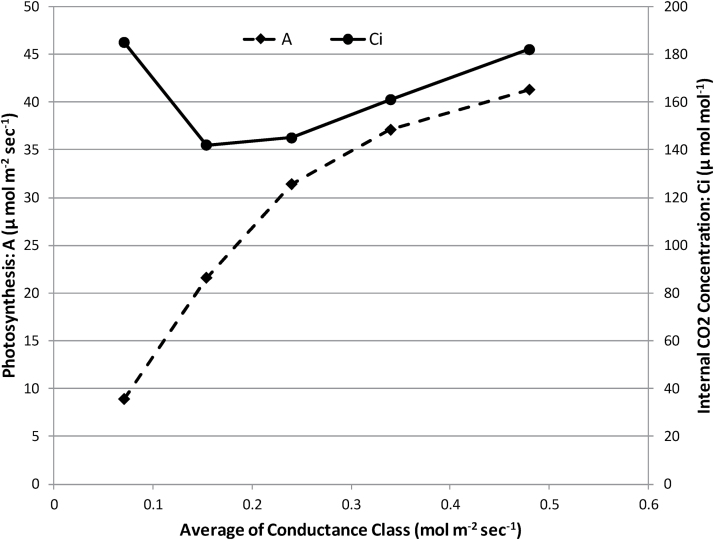
Relationship of photosynthesis (A) and internal leaf CO_2_ concentration (Ci) to conductance. All data have been pooled and averaged for conductance classes as in Table 7.

Genetic variation in TE and Ci were further examined at different levels of conductance and at different times of the day. To examine variation at different levels of conductance, the data were partitioned into five (arbitrary) classes based on conductance levels (mol m^−2^ s^−1^): 0.0–0.1, 0.1–0.2, 0.2–0.3, 0.3–0.4, and >0.4 (and referred to as classes 1 to 5, respectively). An analysis of pooled data with the conductance classes included in the model as a factor indicated there was significant (*P* < 0.001) variation among these classes in photosynthesis and Ci, and also significant variation due to genotype × class interactions. With each step increase in conductance from class 1 to 5, there was a diminishing increase in photosynthesis, so that Ci levels increased as conductance increased above 0.1mol m^−2^ s^−1^ (Table 7, Fig. 3). Mean Ci was relatively high at the lowest level of conductance, indicative of photosynthesis impairment in at least some measurements within that range. Error variance for Ci was particularly large for the lowest conductance class.

**Table 7.** Values of conductance, photosynthesis, and Ci of five classes of gas exchange measurements, where classing of measurements was based on (arbitrary) ranges in conductance levels (measurements with lowest conductance levels in class 1, and those with highest conductance levels in class 5)

Class (number of observations)	Conductance range of group (mean)	Associated measurement	Mean	Variance component due to:		
				Clones	Clones × date	Error
1	0.0–0.1	A	8.9	2.11***	1.57***	13.9
(230)	(0.071)	Ci	185	NS	NS	3284
2	0.1–0.2	A	21.6	1.81***	3.13***	19.2
(946)	(0.154)	Ci	142	75.9***	332***	930
3	0.2–0.3	A	31.4	3.12***	2.20*	8.42
(843)	(0.24)	Ci	145	99.0***	215***	308.2
4	0.3–0.4	A	37.1	5.23***	13.6**	6.99
(299)	(0.34)	Ci	161	164***	355**	185.6
5	>0.4	A	41.3	NS	NS	16.4
(161)	(0.48)	Ci	182	NS	1333**	217.9

For each class of data, the total number of measurements, the range, and mean of conductance levels within the class, and corresponding mean photosynthesis and Ci levels within the class is indicated. Variance components from analyses of A and Ci are indicated. *P < 0.05; **P < 0.01; ***P < 0.001; NS, non-significant.

Within most conductance classes there was significant genetic variation in both photosynthesis and Ci (Table 7). The only exceptions to this were for Ci level in the group with the lowest level of conductance, and for photosynthesis at the highest levels of conductance. For the lowest level of conductance, a lack of genetic variation may be attributable to the very high experimental error variation for Ci.

The mean Ci level of genotypes averaged across all measurements (Table 1) was found to be negatively correlated (*P* < 0.05) with TE on a whole plant basis (Table 8). When phenotypic correlations were examined between the mean Ci level of genotypes within each of the different conductance classes, these were strongest for the middle range of conductance levels, corresponding to class 3 (0.2–0.3mol m^−2^ s^−1^), and lowest for measurements made at the lowest conductance levels. Poor correlation may be expected for the latter owing to the high error variation and the lack of significant genetic variation in Ci at low levels of conductance (Table 7).

**Table 8.** Phenotypic correlations and genetic correlations between TE measured gravimetrically on a whole plant basis and mean Ci of genotypes within each of the five different conductance classes defined in Table 7

	Average Ci	Class1	Class2	Class3	Class4	Class5
Phenotypic	−0.38*	−0.04	−0.18	−0.45**	−0.25*	−0.32*
Genetic	−0.35±0.35	NA	−0.23±0.57	−0.92±0.30	−0.64±0.58	NA
Average Ci	1.00	0.53**	0.69**	0.70**	0.51**	0.43**

Genetic correlations (± standard error) between TE on a whole plant basis and Ci in each class are shown for classes where significant genetic variance existed. The phenotypic correlation between Ci averaged across all measurements and Ci within each class is also shown (third line). *P < 0.05, **P < 0.01, ***P < 0.001 for phenotypic correlations.

Genetic correlations were computed between TEs on a whole plant basis and Ci level was averaged across all the data and for data within each conductance class (Table 8). Only classes for which significant genetic variance existed (classes 2, 3, 4) are meaningful for this statistic and therefore classes 1 and 5 were not considered. Negative genetic correlations were observed for each of classes 2, 3, and 4 but estimates for classes 2 and 4 were associated with high standard errors due to, at least in part, the relatively low broad sense heritability of TE on a whole plant basis (H_b_ = 0.37, Table 2). For the class 3 group of data, there was a strong negative genetic correlation with moderate standard error (−0.92±0.30) observed between Ci and TE on a whole plant basis. This result suggests that leaf level measurements at a mid-level of conductance were the most closely related to and may be of highest predictive value for whole plant TE. Average values of Ci for each genotype within class 3 data are presented in Table 1.

## Discussion

Potentially commercially important genetic variation in TE measured by gravimetric methods was found in a set of sugarcane-related germplasm. This in turn was related partly to intrinsic leaf level TE as estimated by average Ci values. The range in whole plant TE observed across this set of diverse sugarcane genotypes was similar to some previous studies of another C_4_ species, *Sorghum* spp. (Hammer *et al.*, 1997; Xin *et al.*, 2009), with the difference between the lowest and highest TEs being about 40% of the mean (and also about twice the statistical least significant difference value). TE varied across a large range in both commercial-type clones (cultivars and parents in commercial breeding) and wild germplasm clones or close derivatives. The highest value of TE on a whole plant basis observed was for an *Erianthus arundinaceus* clone, which is a wild relative of modern sugarcane cultivars: this species has been of interest to sugarcane breeders for its apparent vigour under water stress (Daniels and Roach, 1987). However, high values were also observed for some cultivars or commercial-type parental clones. These results suggest that progress in breeding programmes for improving this trait may be made if selection for high TE was applied, without necessarily needing to resort to wild germplasm outside of advanced breeding programmes. However, it is also highly likely that much higher levels of TE could be found with more extensive surveys of germplasm both within and outside of commercial breeding programmes, given the relatively small although diverse sampling of germplasm in this study (51 clones) compared with that typically found in germplasm banks (thousands of clones).

Improved TE may provide a profitable target for breeding programmes to improve yields under rainfed or partly irrigated production environments and improve profitability under irrigation. However, understanding the underlying cause of genetic variation in TE is important in determining how it may be used for crop improvement. Several possible mechanisms may be responsible for genetic variation in TE, as indicated in the ‘Introduction’. Mechanisms resulting in concurrent reduced water use and higher TE may lead to net reductions in growth and yield in some environments (Condon *et al.*, 2004; Blum, 2009). In the experiment reported here, TE was slightly negatively correlated with both water use and biomass. Although these relationships were only weak, intense and prolonged selection pressure for TE alone may be expected to reduce biomass production in at least some environments. In this study the three clones with highest TE on a whole plant basis (≥8g/L) had low biomass (and water use) whereas there were some clones with relatively high TE (ca. 7.5g/L) that had relatively large biomass, equivalent to that of clones with much lower TE.

The average Ci/Ca ratio observed across all clones was 0.38 (assuming atmospheric CO_2_ was 390 µmol mol^−1^), similar to that typically observed in other C_4_ species such as sorghum (e.g. 0.33 observed across 30 lines of sorghum by Henderson *et al.*, 1998), which in turn is about half the levels typically observed in C_3_ species (0.7–0.8).

At the leaf level, TE is proportional to the value (1 – Ci/Ca) (Farquhar and Richards, 1984) for any given level of atmospheric VPD. The negative genetic correlation between Ci levels overall and whole plant TE indicates that instantaneous leaf level TE was at least partly responsible for differences in whole plant TE among the genotypes studied. Reduced Ci and therefore higher intrinsic TE may arise owing to either (i) differences among genotypes in average levels of conductance, leading to different positioning of genotypes on the overall curvilinear relationship between conductance and photosynthesis (i.e. high average conductance levels will be associated with higher Ci levels and low TE); or (ii) the higher photosynthesis capacity at any given conductance level. For the relatively narrow range of conductance within each of the five conductance classes, the overall relationship between conductance and photosynthesis would be almost linear, as depicted in Fig. 3, and therefore differences in Ci could be largely attributed to differences in photosynthesis capacity. Genetic variation in Ci at mid-range levels of conductance was found to be reasonably strongly related to gravimetrically estimated whole plant TE than at other levels of conductance, indicating that differences in photosynthetic capacity could also be at least one mechanism causing the observed genetic variation in whole plant TE.

Targeted selection and improvement via breeding of lowered Ci at mid-range levels of conductance may provide longer-term benefits for water-limited environments. Reduced Ci levels at any given level of conductance would be expected to potentially result in improved yields in water-limited environments without large trade-offs in rates of water use and growth. However, it is important to emphasize that these results do not preclude the role of other mechanisms, such as response of conductance to changes in VPD, in also contributing to genetic variation in TE on a whole plant basis in the materials studied. Variation in TE caused by different conductance responses may also play an important role and should be investigated, but are not within the scope of analyses presented in this report.

The relationship between Ci and TE on a whole plant basis was not apparent for data collected at both very low and very high levels of conductance. One possible reason for this is the higher error or environmental variation in Ci at either very low or high levels of conductance, making it more difficult to measure genetic variation precisely. The appearance of a non-stomatal limitation to photosynthesis was evident in conductance class 1 (0–0.1mol m^−2^ s^−1^), as demonstrated by high Ci and comparatively low photosynthesis rates (Table 7). The predominance of non-stomatal limitations at very low conductance levels is widely accepted, and attributed in both C_3_ and C_4_ plants to metabolic inhibition related to factors such as reduced ATP concentration or activity of photosynthesis enzymes, and caused by water stress or damage to leaves (e.g. from wind). These metabolic factors have been extensively reviewed (e.g. Brodribb, 1996; Flexas *et al.*, 2004; Ghannoum, 2009; Lawlor and Tazara, 2009). It would seem unlikely that water stress caused such inhibition in the results reported here because plants were grown under high moisture content and without any apparent water deficits.

Both conductance and photosynthesis were highest in the morning on average, and decreased as the day progressed. This reduction may possibly have been due to mild water stress, but the pots were kept at moisture levels not normally considered likely to result in water stress severe enough to cause damage to photosynthetic machinery. Sink limitations on photosynthesis may also have been a contributing factor (Inman-Bamber *et al.*, 2011).

The rankings and magnitude of genetic effects observed in this study under non-water-stress conditions may differ to those that could occur under water stress due to genotype × water stress interactions associated with either photosynthesis or conductance responses. However, genetic effects for TE under non-stress conditions are important in affecting crop yield in most water-limited environments because a significant proportion of water use during the cycle of crop growth in many production environments, and particularly in sugarcane, occurs under relatively non-stressed conditions. Thus, improved TE at any given biomass growth rate will translate into delayed onset of water stress and thus more sustained growth in water-limited environments. 

Large and potentially important genetic variation in intrinsic TE appears to exist within sugarcane. However, genetic variation in intrinsic TE is just one of the traits that can affect genetic variation in water use efficiency of a crop in the field. Genetic variation in other traits may also play an important role in influencing whole plant TE in the field, such as differences in rate of water use and stomatal conductance over time in response to variation in soil water content (perhaps affected by differences in root growth), different stomatal responses to VPD, and differences in canopy boundary layer conductance. However, genetic variation in TE resulting from different responses in conductance may be prone to significant genotype × environment interactions arising, for example, from root interactions with soil characteristics and climate. While genetic variation in intrinsic TE for any given level of conductance may be only one of a number of sources of variation in crop TE in the field, this study indicates that it could provide a useful target for breeding programmes if reliable and cost-effective screening can be implemented.

## Supplementary Data

Supplementary data are available online at JXB

Table S1. Mean conductance, photosynthesis, and leaf intercellular CO_2_ concentration at each date of measurement.

Supplementary Data
