# Supplementary material for: Genetic variation in transpiration efficiency and relationships between whole plant and leaf gas exchange measurements in Saccharum spp. and related germplasm
Source: J Exp Bot. 2015 Nov 30;67(3):861–71. doi: 10.1093/jxb/erv505 (PMC4737081; doi:10.1093/jxb/erv505)
Supplement: Supplementary Data [file supp_67_3_861__index.html]

Genetic variation in transpiration efficiency and relationships between whole plant and leaf gas exchange measurements in Saccharum spp. and related germplasm — Genetic variation in transpiration efficiency and relationships between whole plant and leaf gas exchange measurements in Saccharum spp. and related germplasm — Supplementary Data 

# Genetic variation in transpiration efficiency and relationships between whole plant and leaf gas exchange measurements in *Saccharum* spp. and related germplasm

## Supplementary Data

Data files

- Supp\_Table\_S1.pdf - Supplementary Data
