# Supplementary material for: Genetic variation in transpiration efficiency and relationships between whole plant and leaf gas exchange measurements in Saccharum spp. and related germplasm
Source: J Exp Bot. 2015 Nov 30;67(3):861–71. doi: 10.1093/jxb/erv505 (PMC4737081; doi:10.1093/jxb/erv505)
Supplement: Supplementary Data [file supp_erv505_Supp_Table_S1.pdf]

Genetic variation in transpiration efficiency in sugarcane, and relationships between whole plant and leaf gas exchange measurements.

Phillip Andrew Jackson, Jaya Basnayake, Geoff Inman-Bamber, Prakash Lakshmanan, Sijesh Natarajan, and Chris Stokes

SUPPLEMENTARY DATA

Supplementary Table S1. Mean conductance, photosynthesis, leaf inter-cellular CO_2 concentration at each date of measurement. Vapour pressure deficit (VPD) and air temperature, averaged over the time which the measurements on each date were taken are also given.

Date	Conductance (mol m^{-2} sec^{-1})	Photosynthesis (μmol m^{-2} sec^{-1})	Ci (μmol mol^{-1})	VPD (kPa)	Temperature (^{0}C)
18 June	0.348	28.79	204.6	1.74	24.3
19 June	0.263	24.08	194.3	1.89	25.3
25 June	0.195	20.69	182.4	2.51	26.4
26 June	0.183	19.84	178.2	1.99	24.4
1st July	0.179	21.00	175.9	2.05	22.4
2nd July	0.156	21.52	139.7	1.61	21.4
5th July	0.261	27.72	171.4	1.23	23.7
10th July	0.182	23.26	140.3	2.24	23.1
14th July	0.203	23.45	163.5	1.37	21.6
16th July	0.279	29.39	165.3	1.61	25.6
17th July	0.281	28.31	166.6	1.79	27.1
21st July	0.190	20.55	179.6	1.59	23.6
4th August	0.247	34.04	116.0	1.86	25.9
5th August	0.254	35.91	114.6	1.61	25.2
6th August	0.368	37.92	152.9	1.68	24.7
7th August	0.204	35.09	86.5	1.63	24.1
8th August	0.233	31.19	149.3	1.61	23.5
13th August	0.175	25.17	150.2	1.75	25.5
14th August	0.253	29.58	156.6	1.39	23.7
19th August	0.171	23.17	145.8	2.24	24.1
20th August	0.193	22.44	166.8	1.88	24.7
21st August	0.193	24.25	160.0	1.83	25.0
22nd August	0.252	29.06	153.9	1.54	24.8
